# Is really epicardial fat a new marker of cardiovascular risk? A case-control cardiac magnetic resonance study

**DOI:** 10.1186/1532-429X-17-S1-P161

**Published:** 2015-02-03

**Authors:** Begoña Igual-Muñoz, Marta Monteagudo, Alicia M Maceira, Inmaculada Salvador, Herminio Morillas, Elena Sanchez, Pilar Sepulveda, Anastasio Montero

**Affiliations:** cardiac image, Eresa, Valencia, Spain; Cardiology, Hospital Peset, Valencia, Spain; Cardiology, Hospital la fe, Valencia, Spain

## Background

The amount of epicardial adipose tissue (EAT) has been associated with the presence of atherosclerotic disease but its role in assessing the risk of obstructive coronary artery disease (CAD) and its relationship with other cardiovascular risk factors is not fully established. We hypothesized that EAT quantified by cardiac magnetic resonance (CMR) is greater in patients with obstructive CAD and could be a new cardiovascular risk marker.

## Methods

To assess the hypothesis, EAT was quantified in patients with ischemia detected in CMR vasodilator stress test and evidence of obstructive CAD in coronary angiogram and also in a group of control patients with similar risk profile but absence of ischemia in CMR stress test and also without diagnosis of obstructive CAD in follow up. Volume of EAT was quantified in 8-10 end-diastolic short axis slices of steady state free precession sequences, derived by Simpson method and indexed by body surface area.

## Results

Seventy patients were included, 46 males (66%), mean age of 60 years (sd: 11.21) of them 42 had obstructive CAD in coronary angiogram. Volume of EAT indexed by body surface area was significantly greater in patients with obstructive CAD :42.43ml/m2 vs 34.35ml/m2, odds ratio: 1,034 [1,001- 1-069 ] p=0.04. Demographic data including cardiovascular risk factors are shown in the table.

**Table 1:** Cardiovascular risk profile in Case-Control groups

	Cases	Controls	P value
Age	64 (SD 11,29)	69 (SD 10,55)	ns
hypertension	28 p (66,7 %)	21 p (75 %)	ns
Diabetes	19 p (45,2 %)	10 p (35,7 %)	ns
Dyslipemia	24 p (57,1 %)	16 p (57,1 %)	ns
Active smoking	12 p (28,6 %)	4 p (14,3 %)	ns
Former smoking	14 p (33,3 %)	5 p (17,9 %)	ns

## Conclusions

We assess the hypothesis that indexed EAT volume is significantly greater in patients with obstructive CAD compared to patients with similar cardiovascular risk profile but without obstructive CAD and could be a promising new cardiovascular risk marker.

## Funding

N/A.

